# Time-series analysis of heart rate and blood pressure in response to changes in work rate before and after 60 days of 6° head down tilt bed rest

**DOI:** 10.1007/s00421-020-04576-2

**Published:** 2020-12-29

**Authors:** Jessica Koschate, L. Thieschäfer, U. Drescher, T. Zieschang, U. Hoffmann

**Affiliations:** 1 Geriatric Medicine, Department for Health Services Research, School of Medicine and Health Sciences, Carl Von Ossietzky University of Oldenburg, Ammerländer Heerstr. 140, 26129 Oldenburg, Germany; 2 Institute of Sport Science, Carl Von Ossietzky University of Oldenburg, Ammerländer Heerstr. 114-118, 26129 Oldenburg, Germany; 3 German Sport University Cologne, Am Sportpark Muengersdorf 6, 50933 Cologne, Germany; 4 Institute of Exercise Training and Sport Informatics, Exercise Physiology, German Sport University Cologne, Am Sportpark Muengersdorf 6, 50933 Cologne, Germany

**Keywords:** Cardiovascular reflex responses, Bed rest, Time series analysis

## Abstract

**Purpose:**

Cardiovascular regulation during exercise, described using time series analysis, is expected to be attenuated after bed rest (BR) and this effect will be dampened by a reactive jumps countermeasure.

**Methods:**

Twenty subjects (29 ± 6 years, 23.6 ± 1.7 kg m^−2^) were tested on a cycle ergometer 9 days (BDC-9) before the beginning of BR as well as 2 (R + 2) and 13 days (R + 13) after the end of BR, applying moderate pseudo-random binary (PRBS) work rate changes. Heart rate (HR) and mean arterial blood pressure (mBP) were measured beat-to-beat and interpolated to 1 s intervals. HR and mBP were cross-correlated [CCF(HR-mBP)] during the PRBS. Eleven subjects participated in a reactive jump countermeasure (JUMP) during the BR period, the other part of the group served as control group (CTRL).

**Results:**

In the CTRL group, significantly lower CCF(HR-mBP) values during BDC-9 were observed compared to R + 2 during the lags 20–25 s and significantly higher values during the lags − 39 s to − 35 s. In the JUMP group, significantly lower CCFs were only observed at R + 2 compared with BDC-9 during the lags 23 s and 24 s, whereas the CCFs for BDC-9 were significantly higher at several lags compared with R + 13.

**Conclusion:**

Attenuations in the regulation of the cardiovascular system during cycling exercise after BR were found in the CTRL group of the RSL study. Cardiovascular regulation in the JUMP group was improved compared to values before the beginning of BR, suggesting the effectiveness of the reactive jumps countermeasure to mitigate the deleterious effects of prolonged BR.

## Introduction

Cardiovascular regulation after changes in work rate (WR), and therefore metabolic demands, is an important process to supply the exercising muscles with oxygen (O_2_). The acute regulations in response to exercise are summarized as ‘baroreflex’ and ‘metaboreflex’ (Fadel 2008; Fisher et al. 2015; Kaufman and Hayes 2002). The primary function of these reflexes is to maintain an adequate blood pressure which ensures a sufficient and economic perfusion of the relevant tissues of the human body during metabolic demands and/or gravity changes and fluid shifts (Fadel 2008). An insufficient baroreflex response is associated with orthostatic intolerance (Blaber et al. 2004). After prolonged bed rest (BR) as well as sojourns in space, orthostatic tolerance and baroreflex responses are attenuated (Pavy-Le Traon et al. 2007).

Changes in baroreflex sensitivity in response to tilts with and without exercise after periods of BR, depended on length of BR (Linnarsson et al. 2006; Sundblad et al. 2000). While no significant alterations in the baroreflex sensitivity during exercise after 42 days of BR were observed, significant decreases in baroreflex sensitivity were found after 120 days of BR, especially during the tilts from a supine to an upright body position combined with dynamic exercise (Linnarsson et al. 2006).

In a randomized controlled trial comparing the effects of reactive jumps in a sledge jump system as a countermeasure during long-term BR (RSL-study) (Kramer et al. 2017a, b), the participants showed slower heart rate (HR) kinetics and a more pronounced blood pressure regulation independent of their participation in the countermeasure (Koschate et al. 2018). In the same study, an increased sympathovagal balance after the BR phase with faster adjustments of the autonomic cardiovascular regulation to tilting sequences in the reactive jump countermeasure group (JUMP) were reported (Maggioni et al. 2018). Linnarsson et al. (2006) described greater fluctuations in mean arterial blood pressure (mBP) as an indicator of a less efficient cardiovascular control during orthostatic challenges. We discussed, that a more distinct mBP response during exercise might be a sign of impaired cardiovascular control as well (Koschate et al. 2018).

Cross-correlations functions (CCFs) between HR and blood pressure have been applied to estimate baroreflex sensitivity at rest (Westerhof et al. 2004). This so-called time series analysis was also used to examine the cardiorespiratory response to changing exercise intensities (Hoffmann et al. 2013).

The analyses (time series analysis) presented in this paper aim at describing the potential effect of the reactive jump countermeasure in the RSL-study on cardiovascular regulation during upright exercise after BR. We hypothesized, that the interaction of HR and mBP, described by CCFs, which was adapted from Westerhof et al. (2004), is attenuated after BR and this effect will be dampened by the applied countermeasure.

## Methods

### Study design

All experiments were approved by the ethics committee of the Northern Rhine Medical Association (Ärztekammer Nordrhein, Düsseldorf, Germany) and were performed in accordance with the ethical standards as laid down in the 1964 Declaration of Helsinki and its later amendments. The detailed framework of the RSL-study was published by (Kramer et al. 2017b). The two campaigns of the BR study were completed in 2015 and 2016 at the ‘:envihab facility’ of the German Aerospace Center in Cologne, Germany. The identical design of both campaigns consisted of 15 days of baseline data collection (BDC), 60 days of head down tilt BR (HDT) and 15 days of reambulation (R +) at the facility. During the campaigns, the subjects participated in various experiments of different investigator groups. The daily schedule was carefully planned, to avoid interferences between the experiments. During the 60 days of BR, a horizontal reactive sledge jump system countermeasure was applied in one half of the participant group (JUMP). The JUMP group performed 48 training sessions during HDT, training duration varied from 8:30 min to up to 17:00 min, according to the four different training sessions (Kramer et al. 2017b). The other half served as control group without any exercise countermeasure (CTRL) during HDT. The participants were allocated to the groups in random order.

### Subjects

Informed consent was obtained from each participant included in the study, ahead of all measurements. The young, healthy participants received a financial reward for their participation.

Of the initial 24 participants, one subject dropped out before the BR period, because of medical reasons unrelated to the study. Two other subjects terminated the BR phase on HDT49 and HDT50, respectively, due to medical reasons, but participated in the recovery phase after the BR period. One (JUMP group) of the two participants who terminated the HDT-phase early could only be tested on a treadmill and the data were not further analyzed in this manuscript. The other subject, who terminated early, was included for statistical analyses, because no differences in comparison with the other participants were observed. The data of two further subjects (CTRL) had to be discarded from the analyses, because the data quality during one of the tests was insufficient for this analysis. Anthropometric data of the subjects included for statistical analyses are shown in Table 1.

**Table 1:** Anthropometric data of all participants

	All [*n* = 20]		JUMP [*n* = 11]		CTRL [*n* = 9]	
	Mean	SD	Mean	SD	Mean	SD
Age [years]						
BDC-9	29	6	29	7	28	6
Height [cm]						
BDC-9	180	6	181	7	179	4
Body mass [kg]						
BDC-9	76.9	7.0	78.5	6.4	75.0	7.9
R + 2	75.3	6.7	77.5	6.6	72.6	6.6
R + 13	75.9	6.7	77.8	6.4	73.5	7.0

*JUMP* countermeasure group, *CTRL* control group

### Assessment of cardiovascular regulation

The kinetics of mBP and HR were tested on an upright cycle ergometer (Excalibur Sport Lode, Groningen, The Netherlands) at BDC-9, R + 2 and R + 13. The WR protocol is described by Koschate et al. (2018) and consisted of 300 s of rest (Rest), 300 s at 30 W (low constant phase; Low), 2 × 300 s sequences of changing WRs (PRBS), 300 s at 80 W (constant phase; High) and 180 s of recovery measurement. Throughout the test, 3 rebreathing maneuvers were recorded to calculate cardiac output (Petrini et al. 1978), which will not be presented in this manuscript. Pedal frequency was kept at 60 rpm during all tests.

HR was assessed beat-to-beat via ECG (Finapres Medical Systems B.V., Amsterdam, The Netherlands). The R-wave intervals of the ECG were analyzed to calculate HR using Matlab 2019a (MathWorks, Natick, Massachusetts, USA). Continuous blood pressure values were obtained beat-to-beat using a finger cuff (Finometer Model 2, Finapres Medical Systems B.V., Amsterdam, The Netherlands). The height correction unit of the Finometer was considered to compensate for the hydrostatic column between finger (placed in a relaxed position on the handle bar of the cycle ergometer) and heart level (BeatScope Easy, Finapres Medical Systems B.V., Amsterdam, The Netherlands). In addition to the cardiovascular data, ventilation (V’_E_) and breathing frequency (BF) were measured breath-by-breath using the Innocor system (Innovision, Odense, Denmark).

### Time series analysis

**Fig. 1:**
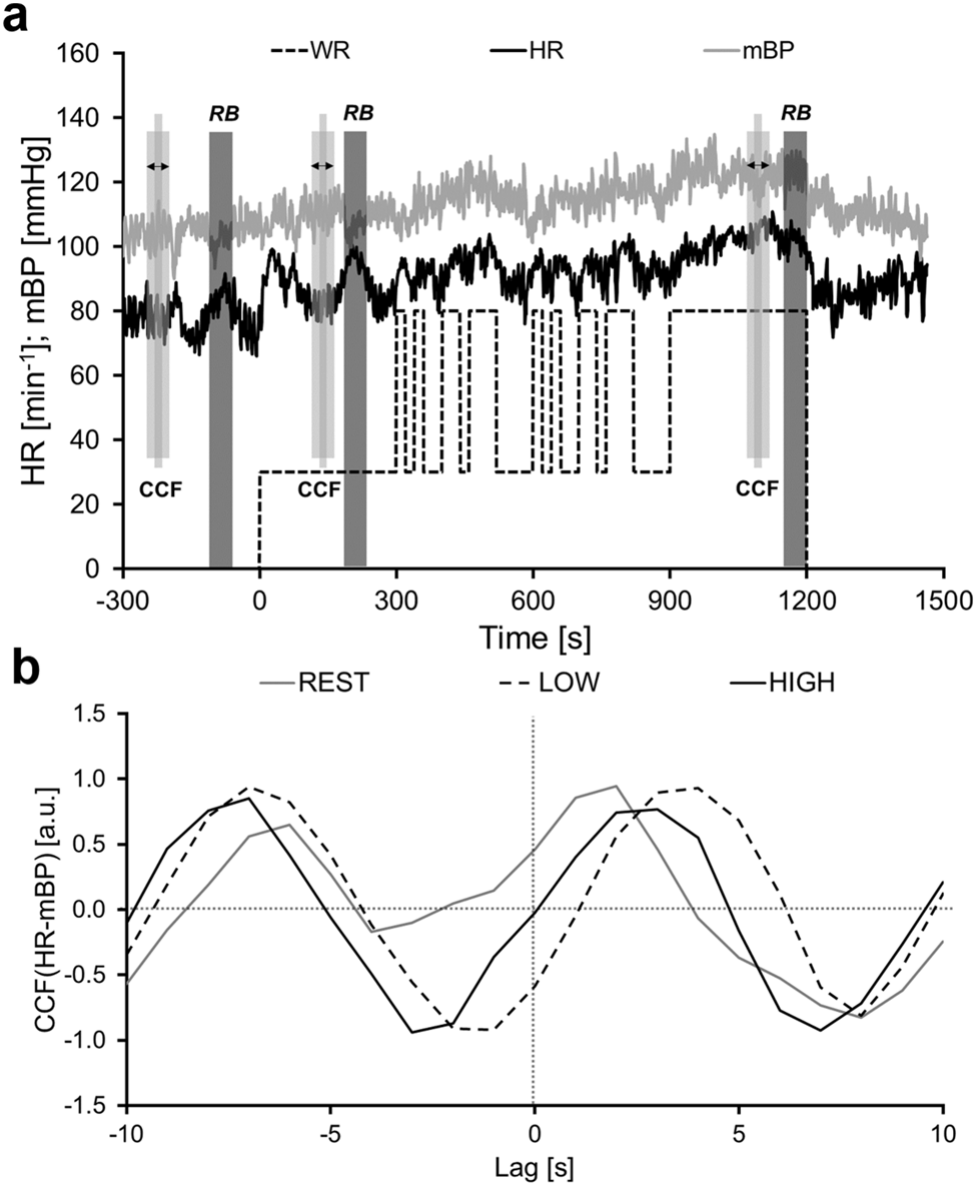
Graphic demonstration of the cross-correlation functions during the steady states. *RB* rebreathing periods, *CCF* cross-correlation functions, *WR* work rate, *HR* heart rate, *mBP* mean blood pressure, *Rest* resting phase, *Low* low constant phase, *High* high constant phase

The data were interpolated to 1 s intervals. During the constant phases of Rest, Low and High short HR sequences of 10 s were shifted 10 s back- and 10 s forward over the mBP signal (Fig. 1) and for each second (lag) the respective CCF(HR-mBP) was calculated (Westerhof et al. 2004). These phases were selected manually considering two criteria: the signal was constant with only small oscillations and no further increase due to change in WR, and the phase was not affected by the rebreathing maneuver (Petrini et al. 1978).

The parameters HR and mBP were chosen instead of inter-beat interval and systolic arterial pressure, because changes in mean arterial blood pressure are more directly proportional to changes in HR and are described as a more reliable estimate of the influence on blood pressure control (Linnarsson et al. 2006). The time frames for the CCF calculations at rest (CCF_rest_) and during the constant WR phases (CCF_Low_, CCF_High_) were chosen as indicated in Fig. 1.

**Fig. 2:**
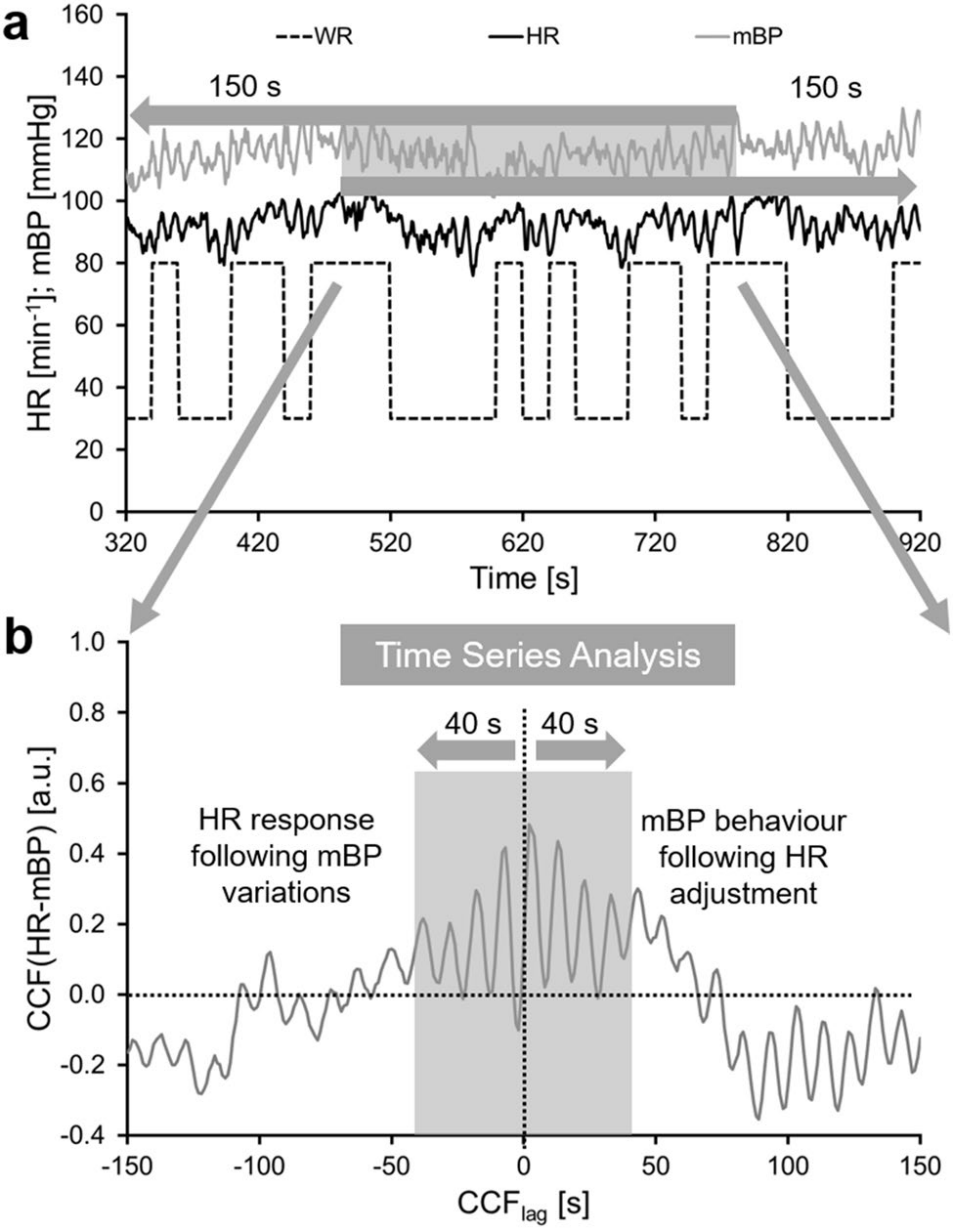
Graphic demonstration of the applied method to describe the baroreflex during the sequences of pseudo randomly changing work rates. *HR* heart rate, *mBP* mean blood pressure, *CCF(HR-mBP)* cross-correlation function of HR and mBP, *CCF*_*lag*_ time shift for the respective cross-correlation function, *a.u.* arbitrary units

For the PRBS data, a 300 s HR interval (see Fig. 2) was shifted 150 s backward and 150 s forward over the mBP signal and a CCF_PRBS_(HR-mBP) was calculated for each shift.

During the constant phases (Fig. 1) and the PRBS (Fig. 2), the negative CCF_lag_ values indicate the backward, and the positive CCF_lag_ values the forward shift of the HR sequence against the mBP values. Positive correlations between HR and mBP at each CCF_lag_ indicate simultaneous increases or decreases of both signals. Negative correlation coefficients indicate an inverse response of the signals. During the exercise phases (Low, PRBS, High), these correlations are also associated with the applied WR. The *backward shift* of the HR sequence against the mBP signal gives information about the mBP behavior before the HR interval that is shifted backwards. Therefore, the HR response to the mBP variations measured before the shifted HR interval is analyzed. A negative value of the CCF indicates that either HR decreases due to an increase in mBP or HR increases due to a decrease in mBP. Corresponding to the backward shift, the *forward shift* yields information about the mBP in response to the variations in the HR interval that is shifted. Negative CCF values indicate that mBP decreases despite an increase in HR, or increases despite a decrease in HR.

For the statistical analyses of the CCF_PRBS_, only the CCFs from − 40 s to 40 s were included (marked in grey in Fig. 2b). This time interval was chosen, since no significant influences from the parameters may occur with a delay of more than 40 s. To compare the CCF_PRBS_ with the data of the constant phases, the CCFs from − 10 s to 10 s were considered, according to the method used by Westerhof et al. (2004).

### Statistical analysis

For a detailed analysis of the regulatory responses between HR and mBP, ANOVA for the time courses of CCF_PRBS_(HR-mBP) from lag − 40 to 40 in 1 s intervals (compare Fig. 2) was calculated with the factors *lag* (− 40–40 s), *group* (CTRL, JUMP), and *day* (BDC-9, R + 2, R + 13). Additionally, the time courses of the CCFs for Rest, Low, PRBS, and High for HR and mBP from lag − 10 s to 10 s in 1 s intervals (compare Figs. 1, 2) were analysed using the factors *lag* (− 10–10 s), *group* (CTRL, JUMP), *day* (BDC-9, R + 2, R + 13), and *step* (Rest, Low, PRBS, High). For the mean values of HR, mBP as well as V’_E_ and BF during the different WR steps, ANOVAs with the factors *day* (BDC-9, R + 2, R + 13), *step* (Rest, Low, PRBS1, PRBS2, High), and *group* (CTRL, JUMP) were calculated, respectively. If sphericity could not be assumed, the Huynh–Feldt test was applied. For post hoc analyses, Bonferroni tests were chosen. The level of significance was set to *p* ≤ 0.05. For all statistical analyses, SPSS 26 (IBM, Amonk, New York, USA) was used.

## Results

The mean values, including significant effects and post hoc results, of HR and mBP during the different phases of the WR protocol are shown in Table 2.

**Table 2:** Mean and standard deviations for HR and mBP during the different WR steps

Parameter	Test day	WR step	All		Post hoc	JUMP		Post hoc	CTRL		Post hoc	Effect/interaction
			Mean	SD		Mean	SD		Mean	SD		
HR	BDC9	Rest	79	12		81	11		77	13	a	Day (*p* < 0.001) Step (*p* < 0.001) Group (*p* = 0.655) Day × group (*p* < 0.001) day × step (*p* < 0.001) Day × step × group (*p* = 0.013) Step × group (*p* = 0.830)
		Low	91	12		93	10		89	13	a	
		PRBS1	101	12	a	103	12		99	12	a	
		PRBS2	105	12	a	107	12		103	13	a	
		High	117	13	a,b	119	13		114	14	a,b	
	R + 2	Rest	82	9		78	8		86	7	c	
		Low	95	10	b	92	9		99	11	b,c	
		PRBS1	108	12	c	104	10		113	14	b,c	
		PRBS2	113	14	c	108	10		120	15	b,c	
		High	131	16	b	125	11		138	19	b,c	
	R + 13	Rest	80	9		79	10		81	7		
		Low	90	9	a	90	9		91	9	a	
		PRBS1	103	9	a	103	8		103	11	a	
		PRBS2	107	9	a	108	7		107	11	a	
		High	122	10	a,c	122	8		121	14	a,c	
mBP	BDC9	Rest	90	15		93	10		86	19		Day (*p* = 0.053) Step (*p* < 0.001) Group (*p* = 0.208) Day × group (*p* = 0.188) Day × step (*p* = 0.031) Step × group (*p* = 0.570) Day × step × group (*p* = 0.226)
		Low	99	14		102	9		94	18		
		PRBS1	100	13		103	8		96	16		
		PRBS2	99	13		104	9		94	16		
		High	99	16	a	104	13		94	19		
	R + 2	Rest	95	12		101	10		88	10		
		Low	103	16		108	17		97	13		
		PRBS1	106	15		111	15		101	14		
		PRBS2	107	15		110	15		103	13		
		High	112	14	c	116	17		108	10		
	R + 13	Rest	93	11		94	9		92	13		
		Low	101	13		99	12		104	14		
		PRBS1	103	14		102	14		104	14		
		PRBS2	102	15		102	16		103	16		
		High	108	14		110	17		106	10		

*HR* heart rate, *mBP* mean blood pressure, *BDC-9* 9 days before the beginning bed rest, R + 2: 2 days after the end of bed rest, R + 13: 13 days after the end of bed rest; a: significantly different to R + 2, b: significantly different to R + 13, c: significantly different to BDC9

Accordingly, mean values of V’_E_ and BF are shown in Table 3.

**Table 3:** Mean and standard deviations for V’_E_ and BF during the different WR steps

Parameter	Test day	WR step	All		Post hoc	JUMP		Post hoc	CTRL		Post hoc		Effect/interaction
			Mean	SD		Mean	SD		Mean	SD			
V’_E_	BDC9	Rest	13.44	2.28		13.49	2.27		14.98	2.39			Day (*p* = 0.385) Step (*p* < 0.001) Group (*p* = 0.825) Day × group (*p* = 0.345) Day × step (*p* = 0.462) Day × step × group (*p* = 0.528) Step × group (*p* = 0.798)
		Low	25.16	2.71		24.73	2.70		27.48	2.84			
		PRBS1	30.96	3.03		30.28	3.01		33.75	3.17			
		PRBS2	32.17	3.20		31.74	3.18		34.84	3.36			
		High	38.71	5.30		38.79	5.28		42.32	5.56			
	R + 2	Rest	13.73	2.71		13.60	2.70		15.74	2.85			
		Low	25.35	3.53		24.81	3.52		28.35	3.71			
		PRBS1	31.42	4.46		31.21	4.44		34.74	4.68			
		PRBS2	32.62	4.26		32.13	4.24		36.08	4.47			
		High	40.80	7.37		39.98	7.33		46.74	7.73			
	R + 13	Rest	13.11	3.21		13.40	3.19		15.05	3.37			
		Low	25.58	4.10		26.74	4.08		27.27	4.30			
		PRBS1	31.21	2.55		31.44	2.54		32.74	2.67			
		PRBS2	32.48	2.93		32.37	2.92		34.63	3.07			
		High	39.27	4.57		39.05	4.55		42.68	4.80			
BF	BDC9	Rest	15.8	2.8		15.3	2.8		16.3	2.9			Day (*p* = 0.648) Step (*p* < 0.001) Group (*p* = 0.549) Day × group (*p* = 0.162) Day × step (*p* = 0.765) Step × group (*p* = 0.680) Day × step × group (*p* = 0.876)
		Low	19.9	3.3		19.0	3.3		20.7	3.5			
		PRBS1	20.7	3.4		19.8	3.4		21.6	3.6			
		PRBS2	21.4	3.7		20.4	3.7		22.4	3.9			
		High	21.8	4.5		21.0	4.5		22.7	4.7			
	R + 2	Rest	15.8	3.4		15.7	3.4		16.0	3.6			
		Low	19.3	4.9		18.7	4.9		19.9	5.1			
		PRBS1	20.6	4.8		19.6	4.8		21.7	5.0			
		PRBS2	21.2	4.7		19.9	4.7		22.5	4.9			
		High	21.4	5.2		21.1	5.2		21.8	5.4			
	R + 13	Rest	14.8	5.4		14.9	5.4		14.8	5.7			
		Low	19.6	4.4		20.0	4.4		19.2	4.6			
		PRBS1	20.4	3.7		20.3	3.7		20.6	3.9			
		PRBS2	21.0	3.9		20.5	3.9		21.4	4.1			
		High	21.7	5.1		21.8	5.0		21.6	5.3			

*V’*_*E*_ ventilation, *BF* breathing frequency, *BDC-9* 9 days before the beginning bed rest, R + 2: 2 days after the end of bed rest, R + 13: 13 days after the end of bed rest

**Fig. 3:**
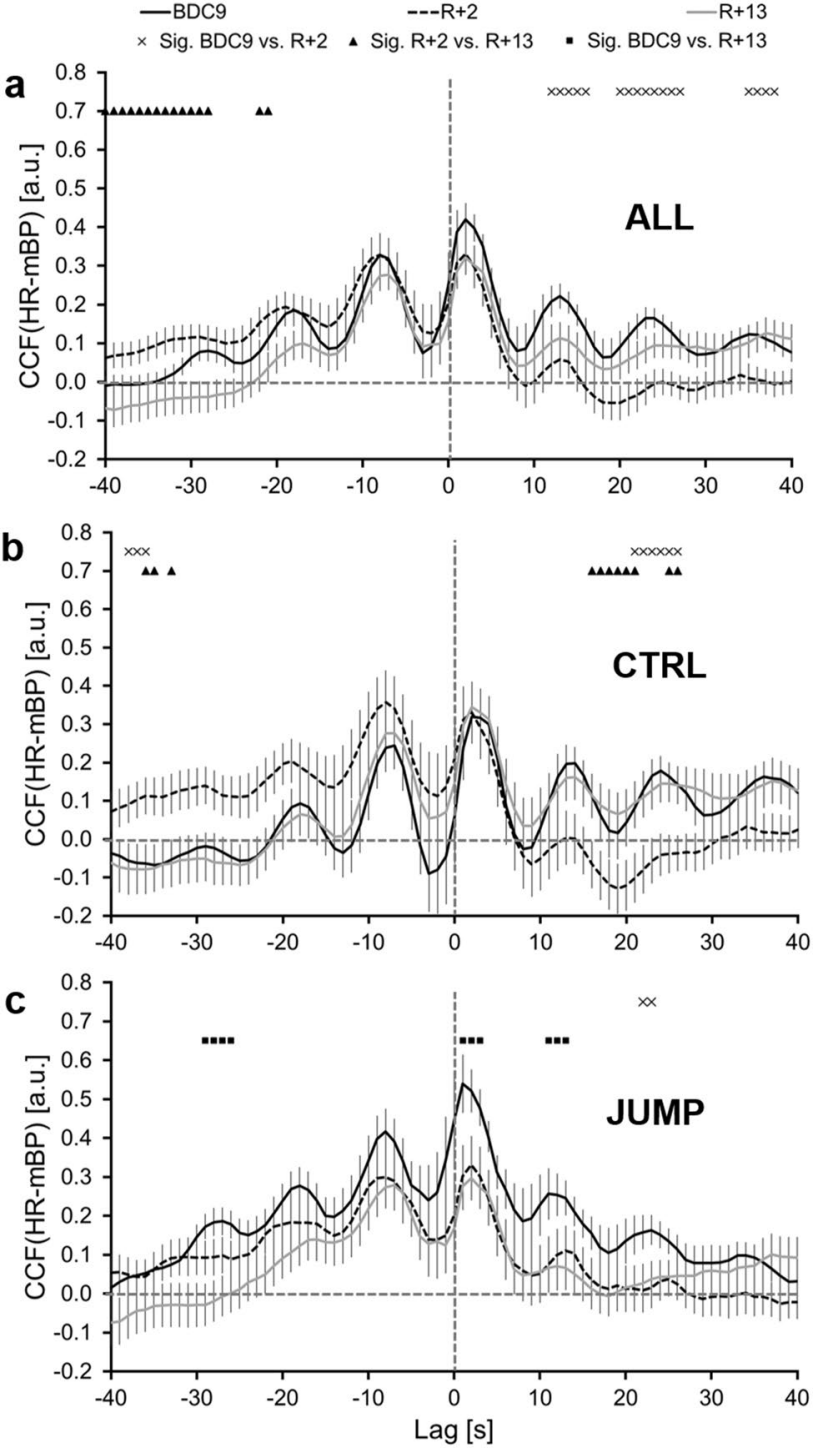
Means (± SE) of CCF (HR-mBP) during the PRBS in comparison between the different study days for the entire group and CTRL and JUMP. BDC-9: nine days before the bed rest phase, R + 2: two days after the bed rest phase, R + 13: thirteen days after the bed rest phase, *CCF* cross-correlation function, *HR* heart rate, *mBP* mean blood pressure, *Sig.* significant difference, *CTRL* control group, *JUMP* countermeasure group

The ANOVA considering the CCF_PRBS_(HR-mBP) values for day × lag × group revealed a significant main effect for lag (*p* < 0.001), and significant effects for the interactions of day × lag (*p* < 0.001) and day × lag × group (*p* = 0.030). No significant effects were found for day, group, day × group, or lag × group. Post hoc results for day × lag × group are shown in Fig. 3.

In addition to the post hoc results shown in Fig. 3, significantly higher CCFs for JUMP were documented for several lags compared with CTRL at BDC-9 (p = 0.042, lags: − 37 s to − 9 s, − 4 s to 3 s, 6 s, 38 s and 39 s). In Fig. 3a, a significantly lower CCF(HR-mBP) of the forward shift in the entire group (ALL: 10–40 s) at R + 2 compared with BDC9 at several lags is shown. This is independent of the applied reactive jumps countermeasure. The CCF(HR-mBP) resulting from the *backward shift*, was significantly lower at R + 13 compared with R + 2 (lags − 40 s to − 20 s).

In the CTRL group (Fig. 3b), significantly lower CCF(HR-mBP) during BDC-9 was observed compared to R + 2 during the *forward shift* (lag 20–25 s) and significantly higher values during the backward shift (− 39 to − 35 s). In similar time frames, the CCFs at R + 2 were significantly different to the CCFs at R + 13, comparable to the difference between BDC-9 and R + 2.

In the JUMP group, significantly lower CCFs were only observed at R + 2 compared with BDC-9 during the forward shift (lag 23 s and 24 s). The CCFs for R + 13 were significantly lower at several lags during the forward and backward shift compared with BDC-9. No significant differences between R + 2 and R + 13 were observed in this group.

**Fig. 4:**
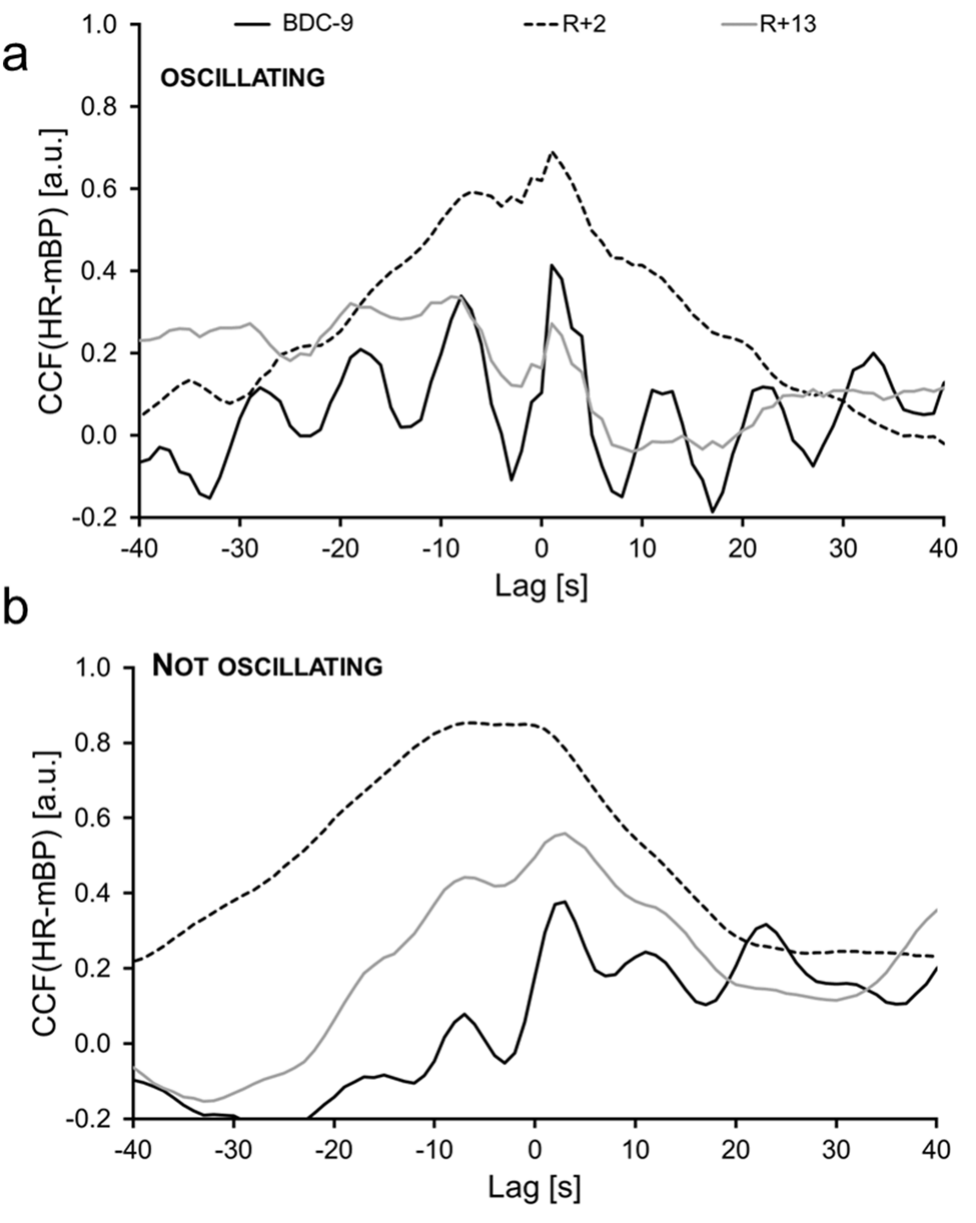
Representative CCF(HR-mBP) of one participant with (a) and one without (b) oscillations during the PRBS for the different study days. BDC-9: nine days before the bed rest phase, R + 2: two days after the bed rest phase, R + 13: thirteen days after the bed rest phase, *CCF* cross-correlation function, *HR* heart rate; *mBP* mean blood pressure

Interestingly, different types of CCFs could be identified (Fig. 4). Some of the participants showed a frequent oscillating behavior for the CCF at BDC9, which was attenuated at R + 2 and began oscillating again at R + 13 (Fig. 4a). In other participants, only small oscillations in the CCFs could be identified (Fig. 4b). An exploratory analysis did not show any differences in cardiorespiratory or anthropometric parameters between the different groups.

**Fig. 5:**
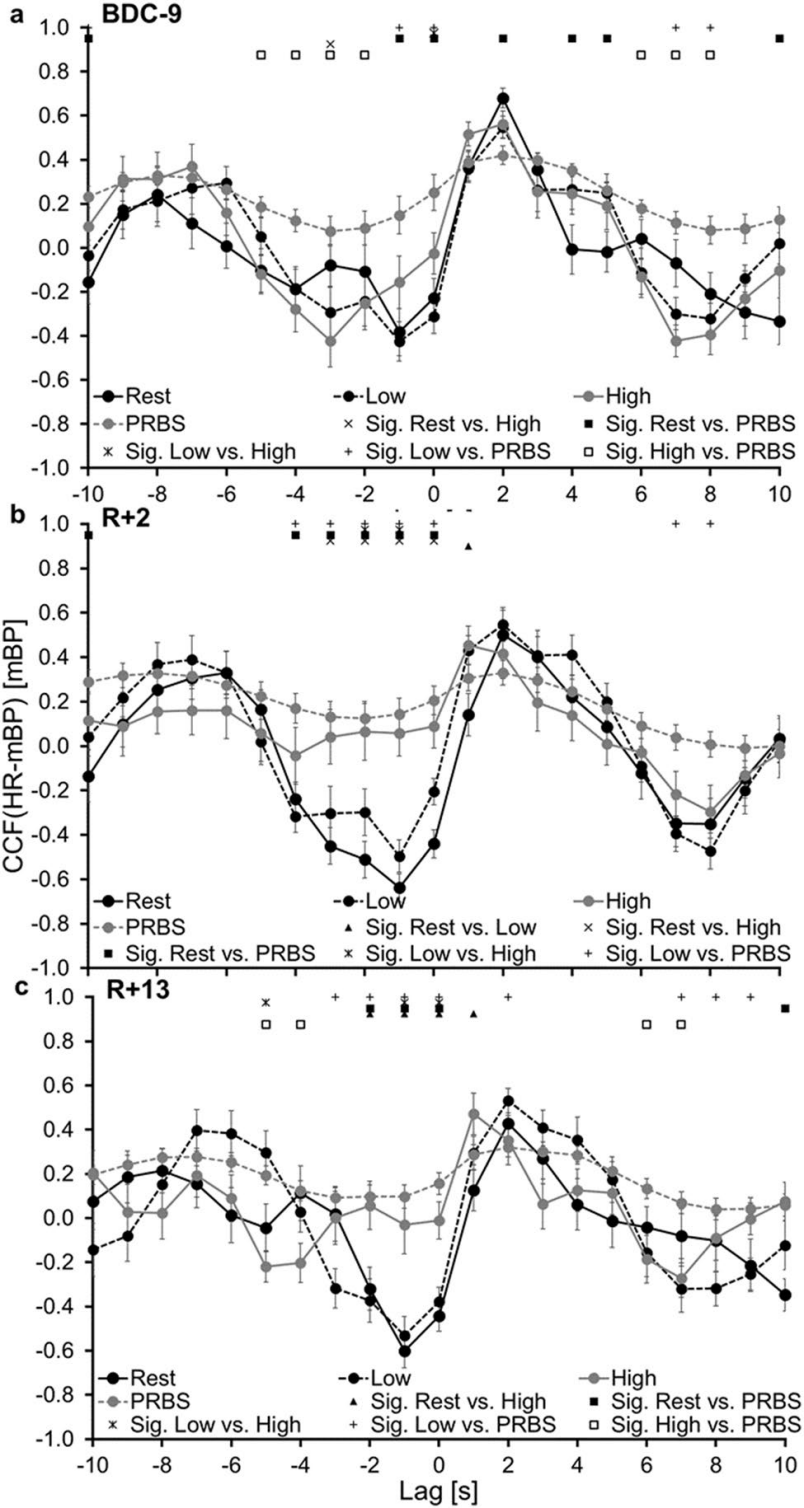
Means (± SE) of the cross-correlation function between heart rate (HR) and mean blood pressure (mBP) during the different exercise intensities at the different test days. **a** Nine days before the beginning of the bed rest period (BDC-9), **b** Two days after the end of bed rest (R + 2); **c** Thirteen days after the end of bed rest (R + 13). *Rest* resting phase, *Low* 30 W constant phase, *High* 80 W constant phase, *Mid* changing work rate of 30 W and 80 W, *CCF* cross-correlation function, *Sig.* significant difference

**Fig. 6:**
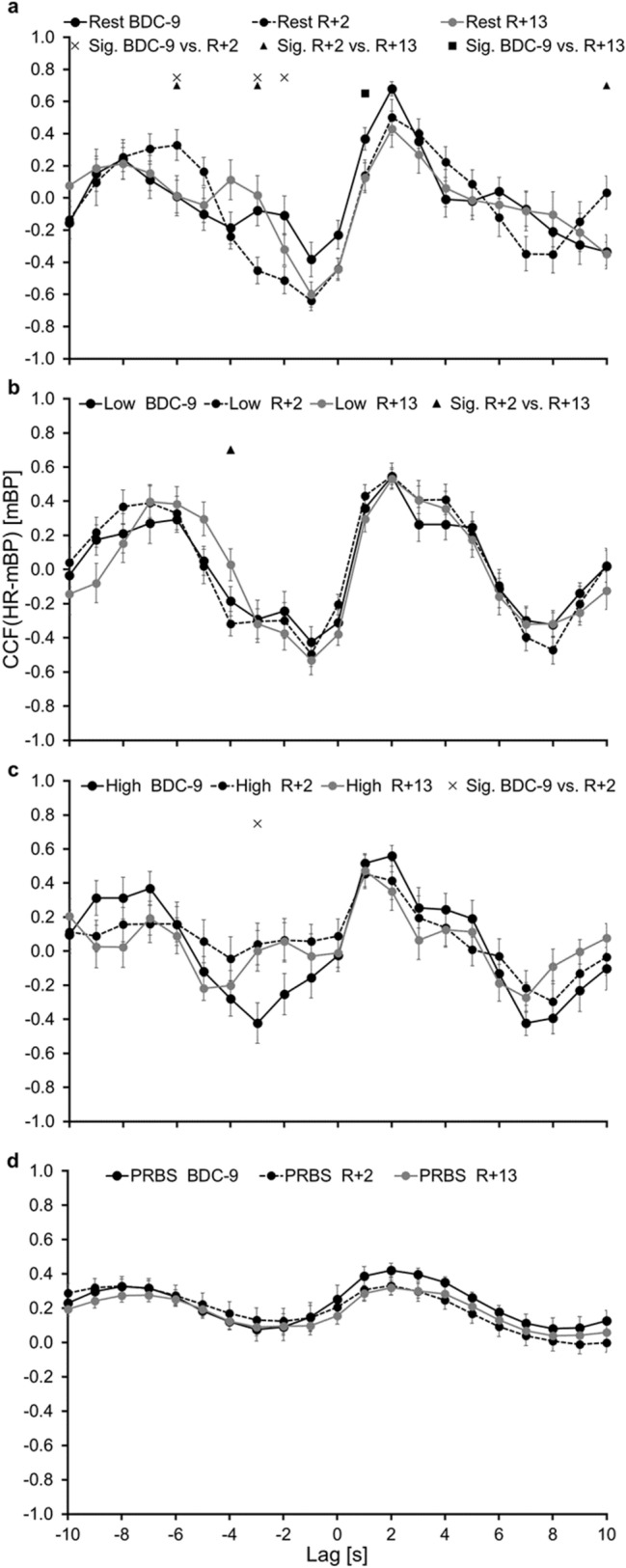
Means (± SE) of the cross-correlation function between heart rate (HR) and mean blood pressure (mBP) for the different test days, compared during the different exercise intensities **a** resting phase (Rest), **b** 30 W constant phase (Low), **c** 80 W constant phase (High), **d** changing work rates of 30 W and 80 W (PRBS). BDC-9: nine days before the bed rest phase, R + 2: two days after the bed rest phase, R + 13: thirteen days after the bed rest phase, *CCF* cross-correlation function, *Sig.* significant difference

ANOVA for day × lag × step × group including the constant phase CCFs (compare Fig. 1), revealed a significant effect for lag (*p* < 0.001), step (*p* < 0.001), as well as day × lag × step (*p* = 0.020) and no significant effect for the factor group. Post hoc results are shown in Figs. 5, 6.

## Discussion

The aim of these analyses was to describe potential changes of cardiovascular regulation during upright exercise following 60 d of BR with and without a reactive jumps countermeasure, applying time series analysis. The hypothesis of an attenuated cardiovascular regulation has been partially substantiated for the CTRL group, but not for the JUMP group. This provides an indication of a positive effect of the applied reactive jumps countermeasure for cardiovascular regulation and emphasizes the practicability of the applied data analysis.

Considering the CCF_PRBS_ immediately after the BR phase (R + 2), the mBP response to variations in HR (forward shift of the HR sequence) as well as the HR response to mBP variations was attenuated in the CTRL group (Fig. 3b). These attenuations disappeared after a recovery period of 11 days (R + 13). In contrast, in the JUMP group (Fig. 3c) at R + 13, significantly lower CCF_PRBS_ during the forward and backward shifts was observed compared with BDC-9, indicating an improvement in cardiovascular regulation and therefore a combined effect of the countermeasure and the recovery period after the BR phase.

Comparisons between the two groups at the different test days (compare Fig. 3b, c) showed significantly stronger HR responses to variations in mBP in the JUMP group at several lags during the backward shift before (BDC-9) but not after the BR period (R + 2 and R + 13). This indicates less adequate HR reactions to variations in mBP in the JUMP group compared with CTRL before the BR phase. Since this difference is diminished after BR, these findings further substantiate the effectiveness of the countermeasure to protect the cardiovascular regulation from deteriorations during BR. The training did not only preserve, but improve the cardiovascular regulation of the JUMP group throughout the BR phase. This is in line with other findings of this RSL-study (Maggioni et al. 2018; Kramer et al. 2017a, b). During rest, enhancements in vagal tone in the JUMP group compared to the CTRL group of the RSL study and an increased sympathovagal balance after the BR phase were found (Maggioni et al. 2018). Additionally, faster responses of the autonomic cardiovascular system to changes in posture in the JUMP group were reported (Maggioni et al. 2018). Considering the mean values of the CTRL group during the different WR steps of the exercise protocol (Table 2), a significantly higher HR at R + 2 compared with BDC-9 was observed during all WR steps and during Rest, which is in accordance with the results of Maggioni et al. (2018). However, except for the High phase, this effect was diminished at R + 13. For the JUMP group, no changes in HR or mBP were documented.

Comparing the CCFs of the different WR steps from lag − 10 s to 10 s, an effect of the WR intensity is visible. At R + 2, no significant difference was observed between CCF_PRBS_ and CCF_High_. In contrast, significant differences were found for BDC-9 and albeit to a lesser extent still at R + 13 (Figs. 5, 6). These findings suggest that especially at the WR intensity of 80 W changes in HR and mBP interaction as a result of the BR period are visible. Potentially, greater differences in the regulation between HR and mBP during exercise in response to BR might be visible at higher WR intensities.

The greatest changes in mBP in response to exercise typically occur after 6–8 s resulting from a delay time of change in muscle sympathetic nerve activity and the subsequent change in vascular resistance and therefore arterial blood pressure (Fadel 2008). This might explain the frequent oscillations of the CCF(HR-mBP) signal during the PRBS in approximately 8 s intervals. An adequate regulation of the cardiovascular system during rest should be represented by oscillations of the CCF(HR-mBP) response around zero. In accordance with the resetting of the baroreflex set point during exercise, the CCF oscillations might be shifted to negative or positive values during the interval of − 20 s to 20 s, as this interval can be interpreted as a WR impulse according to the PRBS WR protocol with 20 s stimuli (Hoffmann et al. 2013; Bennett et al. 1981).

During exercise, several mechanisms of the cardiovascular regulation should be considered: central command, metaboreflex, feedback from ergoreceptors in the working muscle, and baroreceptor resetting (Michelini et al. 2015). A tight balance between the carotid baroreceptor control of the vascular system and the exercise-induced inhibition of sympathetic influences in the active skeletal muscle is important to ensure the adequate regulation of skeletal muscle blood flow to meet the metabolic demands of the exercising muscle and the continued regulation of blood pressure at the same time (Fadel 2008). The vascular system of the non-exercising muscles and visceral organs has to be adequately constricted to redistribute the cardiac output to the active skeletal muscles. At the same time, the metaboreflex causes an attenuation of vasoconstriction in the active tissue (skeletal muscle), which is linked to the accumulation of metabolic substances. Therefore, the autonomic nervous system plays a critical role in mediating the cardiovascular adjustments necessary to meet the metabolic demands of the exercising muscle, and as such is paramount for the performance and sustainment of physical activity (Hansen et al. 2009; Fadel 2008; Fisher et al. 2015; Kaufman and Hayes 2002). Faster cardiorespiratory kinetics during a PRBS-test were already associated with advantageous adjustments in HR during orthostatic stress (Koschate et al. 2019). Changes in the regulation of the cardiovascular system as observed for the interaction of mBP and HR after BR in the CTRL group might, therefore, influence physical fitness, as observed in the parameter V’O_2peak_ for the CTRL group of the RSL study (Kramer et al. 2017a; Koschate et al. 2018). However, the influence of this attenuated cardiovascular regulation might be different for moderate, high and maximal exercise intensities, since no changes in muscular oxygen uptake kinetics were found in the RSL participants after the BR period (Koschate et al. 2018) and as the presented results for the different WR phases suggest (Fig. 5 & 6).

The decrease in the regulation of the cardiovascular system after BR without a countermeasure in response to changing exercise intensities in the upright posture on a cycle ergometer is comparable to the results of other long-term BR interventions (Linnarsson et al. 2006; Maggioni et al. 2018) after 120 days and 60 days of BR, but not to those after 42 days of BR (Sundblad et al. 2000). In the three references, passive tilts with and without exercise were used to analyze cardiovascular regulation. The presented results indicate, that cardiovascular regulation is not only altered during passive tilts after 60 and 120 days of BR but the changes are also detectable during WR changes without tilting the participant up and down.

The impairments may be caused by alterations at the levels of the baroreceptors, the central processing of afferent signals, or in the effector organs (Linnarsson et al. 2006). During the reactive jump countermeasure, the head and therefore the carotid baroreceptor might have been transiently above the aortic baroreceptor, depending on the individual strategy for the jumping movement. This might have challenged the cardiovascular system with a fluid gradient more similar to the demands in the upright posture and might at least in part explain the effectiveness of the countermeasure in the JUMP group. Additionally, Kramer et al. (2017a) found the jump training intervention to prevent changes in V’O_2peak_, maximal leg strength, and lean muscle mass, in the JUMP, but not the CTRL group after bed rest, the latter possibly with a beneficial effect on the functioning of the muscle pump in the working muscle.

An enhanced baroreceptor sensitivity, which would be represented by a pronounced response of HR to mBP changes in the applied analysis, was described as an indicator of parasympathetic predominance (Reynolds et al. 2017). The results of the time series analysis indicate a reduced parasympathetic predominance, due to an attenuated response of HR to mBP changes. In this context, an impaired cardiac acceleration during exercise after BR, especially regarding the range controlled by sympathetic activity was discussed (Linnarsson et al. 2006).

It is also argued, that the primary purpose of the systemic circulation is to provide adequate perfusion to all the various tissues of the body under conditions of varying metabolic needs and different postures. Arterial blood pressure is reported as the most tightly controlled variable in the human cardiovascular system and should therefore be the most relevant one to be analyzed. Greater amplitudes in arterial pressure in response to orthostatic stress would indicate a less efficient cardiovascular control (Linnarsson et al. 2006). In the context of the time series analysis during randomly changing work rate intensities, very positive or negative values are an indicator of an inefficient regulation or the resetting of the baroreceptors during exercise. The primary mechanism responsible for the baroreflex resetting during exercise is the progressive vagal withdrawal at exercise onset (Raven et al. 2006). In this regard, independent of the applied reactive jumps countermeasure, slowed HR kinetics in combination with higher HR values during the different WR steps of the exercise protocol were documented after BR for the RSL study (Koschate et al. 2018). This presumably greater proportion in sympathetic system activity of HR control during exercise might explain the changes in the interplay of HR and mBP.

V’_E_ and BF, as potential influencing factors on blood pressure regulation did not change throughout the study. Therefore, the changes in cardiovascular regulation cannot be explained by changes in mean values of these parameters. However, in future analyses, CCFs between V’_E_ and BF should be calculated to obtain more detailed insights in the mechanisms leading to the observed changes in the cardiovascular system.

## Limitations

Relative exercise intensity was greater at R + 2 compared with BDC-9 (Koschate et al. 2018). Since baroreflex resetting is directly linear to exercise intensity (Fadel 2008), the baroreflex was potentially reset to a greater extent at R + 2 compared with BDC-9.

The significant differences between the test days were mostly located at lags not proximate to the WR impulse. Therefore, the regulation during exercise might not be affected severely, but the return to baseline after the WR stimulus might be delayed.

Since a significant difference between JUMP and CTRL was observed at BDC-9, the results of the entire group, as presented in Fig. 3a should be interpreted with caution. It seems that the CTRL group had a better cardiovascular regulation before the BR period.

The CCF analysis was applied as a tool for the detection of delayed reactions. Since no dynamic linearity in terms of system control can be assumed, no quantitative regulation model can be derived from these results. This should be considered for the interpretation of the results. However, the data are not interpreted statistically for a correlation with statistical significance, but for identifying similar patterns of the parameters in response to the applied WR stimuli.

The distinctive degree of oscillations between the different participants and the study days complicate the comparability. This effect should be further analyzed using sophisticated methods to determine the reason for different degrees of oscillations.

## Conclusion

Attenuations in the regulation of the cardiovascular system during upright cycling exercise after BR were found in the CTRL group of the RSL study. Cardiovascular regulation in the JUMP group was improved compared to values before the beginning of the BR period, suggesting the effectiveness of the reactive jumps countermeasure to mitigate the deleterious effects of prolonged BR. To date, changes in the acute regulation of HR and mBP after BR were only shown for tilts with and without exercise, but not for changing WRs in a stable, upright body position. Using time series analyses, it was possible to show the changes in cardiovascular regulation during exercise for the first time without tilting and in the moderate exercise intensity range, which can easily be applied without exhaustive effort of the participant. Additionally, the test enables to measure cardiorespiratory regulation as an indicator of fitness and at the same time cardiovascular reflex responses.

This analysis should also be tested for the comparison of patients with cardiovascular diseases with healthy adults, although it might be necessary to lower exercise intensity further for some patient groups, especially those having bed rest due to medical conditions. Using the forward and backward shift of HR against mBP, the respective responses of the parameters can be analyzed in detail.

## Abbreviations

ANOVA: Analysis of variance
BDC: Baseline data collection
BR: Bed rest
CCF_lag_: Lag in cross-correlation function
CCF: Cross correlation function
CTRL: Control group
HDT: Head down tilt phase
High: High constant phase
HR: Heart rate
JUMP: Reactive jumps countermeasure group
Low: Low constant phase
mBP: Mean blood pressure
O_2_: Oxygen
PRBS: Pseudo-random binary sequences
R +: Recovery period
Rest: Resting phase
WR: Work rate
